# Natural language processing algorithms for mapping clinical text fragments onto ontology concepts: a systematic review and recommendations for future studies

**DOI:** 10.1186/s13326-020-00231-z

**Published:** 2020-11-16

**Authors:** Martijn G. Kersloot, Florentien J. P. van Putten, Ameen Abu-Hanna, Ronald Cornet, Derk L. Arts

**Affiliations:** 1 Amsterdam UMC, University of Amsterdam, Department of Medical Informatics, Amsterdam Public Health Research Institute Castor EDC, Room J1B-109, PO Box 22700, 1100 DE Amsterdam, The Netherlands; 2 Castor EDC, Amsterdam, The Netherlands

**Keywords:** Ontologies, Entity linking, Annotation, Concept mapping, Named-entity recognition, Natural language processing, Evaluation studies, Recommendations for future studies

## Abstract

**Background:**

Free-text descriptions in electronic health records (EHRs) can be of interest for clinical research and care optimization. However, free text cannot be readily interpreted by a computer and, therefore, has limited value. Natural Language Processing (NLP) algorithms can make free text machine-interpretable by attaching ontology concepts to it. However, implementations of NLP algorithms are not evaluated consistently. Therefore, the objective of this study was to review the current methods used for developing and evaluating NLP algorithms that map clinical text fragments onto ontology concepts. To standardize the evaluation of algorithms and reduce heterogeneity between studies, we propose a list of recommendations.

**Methods:**

Two reviewers examined publications indexed by Scopus, IEEE, MEDLINE, EMBASE, the ACM Digital Library, and the ACL Anthology. Publications reporting on NLP for mapping clinical text from EHRs to ontology concepts were included. Year, country, setting, objective, evaluation and validation methods, NLP algorithms, terminology systems, dataset size and language, performance measures, reference standard, generalizability, operational use, and source code availability were extracted. The studies’ objectives were categorized by way of induction. These results were used to define recommendations.

**Results:**

Two thousand three hundred fifty five unique studies were identified. Two hundred fifty six studies reported on the development of NLP algorithms for mapping free text to ontology concepts. Seventy-seven described development and evaluation. Twenty-two studies did not perform a validation on unseen data and 68 studies did not perform external validation. Of 23 studies that claimed that their algorithm was generalizable, 5 tested this by external validation. A list of sixteen recommendations regarding the usage of NLP systems and algorithms, usage of data, evaluation and validation, presentation of results, and generalizability of results was developed.

**Conclusion:**

We found many heterogeneous approaches to the reporting on the development and evaluation of NLP algorithms that map clinical text to ontology concepts. Over one-fourth of the identified publications did not perform an evaluation. In addition, over one-fourth of the included studies did not perform a validation, and 88% did not perform external validation. We believe that our recommendations, alongside an existing reporting standard, will increase the reproducibility and reusability of future studies and NLP algorithms in medicine.

**Supplementary Information:**

**Supplementary information** accompanies this paper at 10.1186/s13326-020-00231-z.

## Background

One of the main activities of clinicians, besides providing direct patient care, is documenting care in the electronic health record (EHR). Currently, clinicians document clinical findings and symptoms primarily as free-text descriptions within clinical notes in the EHR since they are not able to fully express complex clinical findings and nuances of every patient in a structured format [1, 2]. These free-text descriptions are, amongst other purposes, of interest for clinical research [3, 4], as they cover more information about patients than structured EHR data [5]. However, free-text descriptions cannot be readily processed by a computer and, therefore, have limited value in research and care optimization.

One method to make free text machine-processable is entity linking, also known as annotation, i.e., mapping free-text phrases to ontology concepts that express the phrases’ meaning. Ontologies are explicit formal specifications of the concepts in a domain and relations among them [6]. In the medical domain, SNOMED CT [7] and the Human Phenotype Ontology (HPO) [8] are examples of widely used ontologies to annotate clinical data. After the data has been annotated, it can be reused by clinicians to query EHRs [9, 10], to classify patients into different risk groups [11, 12], to detect a patient’s eligibility for clinical trials [13], and for clinical research [14].

Natural Language Processing (NLP) can be used to (semi-)automatically process free text. The literature indicates that NLP algorithms have been broadly adopted and implemented in the field of medicine [15, 16], including algorithms that map clinical text to ontology concepts [17]. Unfortunately, implementations of these algorithms are not being evaluated consistently or according to a predefined framework and limited availability of data sets and tools hampers external validation [18].

To improve and standardize the development and evaluation of NLP algorithms, a good practice guideline for evaluating NLP implementations is desirable [19, 20]. Such a guideline would enable researchers to reduce the heterogeneity between the evaluation methodology and reporting of their studies. Generic reporting guidelines such as TRIPOD [21] for prediction models, STROBE [22] for observational studies, RECORD [23] for studies conducted using routinely-collected health data, and STARD [24] for diagnostic accuracy studies, are available, but are often not used in NLP research. This is presumably because some guideline elements do not apply to NLP and some NLP-related elements are missing or unclear. We, therefore, believe that a list of recommendations for the evaluation methods of and reporting on NLP studies, complementary to the generic reporting guidelines, will help to improve the quality of future studies.

In this study, we will systematically review the current state of the development and evaluation of NLP algorithms that map clinical text onto ontology concepts, in order to quantify the heterogeneity of methodologies used. We will propose a structured list of recommendations, which is harmonized from existing standards and based on the outcomes of the review, to support the systematic evaluation of the algorithms in future studies.

## Methods

This study consists of two phases: a systematic review of the literature and the formation of recommendations based on the findings of the review.

### Literature review

A systematic review of the literature was performed using the Preferred Reporting Items for Systematic reviews and Meta-Analyses (PRISMA) statement [25].

### Search strategy and study selection

We searched Scopus, IEEE, MEDLINE, EMBASE, the Association for Computing Machinery (ACM) Digital Library, and the Association for Computational Linguistics (ACL) Anthology for the following keywords: Natural Language Processing, Medical Language Processing, Electronic Health Record, reports, charts, clinical notes, clinical text, medical notes, ontolog*, concept*, encod*, annotat*, code, and coding. We excluded the words ‘reports’ and ‘charts’ in the ACL and ACM databases since these databases also contain publications on non-medical subjects. The detailed search strategies for each database can be found in Additional file 2. We searched until December 19, 2019 and applied the filters “English” and “has abstract” for all databases. Moreover, we applied the filters “Medicine, Health Professions, and Nursing” for Scopus, the filters “Conferences”, “Journals”, and “Early Access Articles” for IEEE, and the filter “Article” for Scopus and EMBASE. EndNote X9 [26] and Rayyan [27] were used to review and delete duplicates.

The selection process consisted of three phases. In the first phase, two independent reviewers with a Medical Informatics background (MK, FP) individually assessed the resulting titles and abstracts and selected publications that fitted the criteria described below.

Inclusion criteria were:

- Medical language processing as the main topic of the publication
- Use of EHR data, clinical reports, or clinical notes
- Algorithm performs annotation
- Publication is written in English

Some studies do not describe the application of NLP in their study by only listing NLP as the used method, instead of describing its specific implementation. Additionally, some studies create their own ontology to perform NLP tasks, instead of using an established, domain-accepted ontology. Both approaches limit the generalizability of the study’s methods. Therefore, we defined the following exclusion criteria:

- Implementation was not described
- Implementation does not use an existing established ontology for encoding
- Not published in a peer-reviewed journal (except for ACL and ACM publications)

In the second phase, both reviewers excluded publications where the developed NLP algorithm was not evaluated by assessing the titles, abstracts, and, in case of uncertainty, the Method section of the publication. In the third phase, both reviewers independently evaluated the resulting full-text articles for relevance. The reviewers used Rayyan [27] in the first phase and Covidence [28] in the second and third phases to store the information about the articles and their inclusion. In all phases, both reviewers independently reviewed all publications. After each phase the reviewers discussed any disagreement until consensus was reached.

### Data extraction and categorization

Both reviewers categorized the implementations of the found algorithms and noted their characteristics in a structured form in Covidence. The objectives of the included studies and their associated NLP tasks were categorized by way of induction. The results were compared and merged into one result set.

We collected the following characteristics of the studies, based on a combination of TRIPOD [21], STROBE [22], RECORD [23], and STARD [24] statement elements (see Additional file 3): year, country, setting, objectives, evaluation methods, used NLP systems or algorithms, used terminology systems, size of datasets, performance measures, reference standard, language of the free-text data, validation methods, generalizability, operational use, and source code availability.

### List of recommendations

Based on the findings of the systematic review and elements from the TRIPOD, STROBE, RECORD, and STARD statements, we formed a list of recommendations. The recommendations focus on the development and evaluation of NLP algorithms for mapping clinical text fragments onto ontology concepts and the reporting of evaluation results.

## Results

**Fig. 1:**
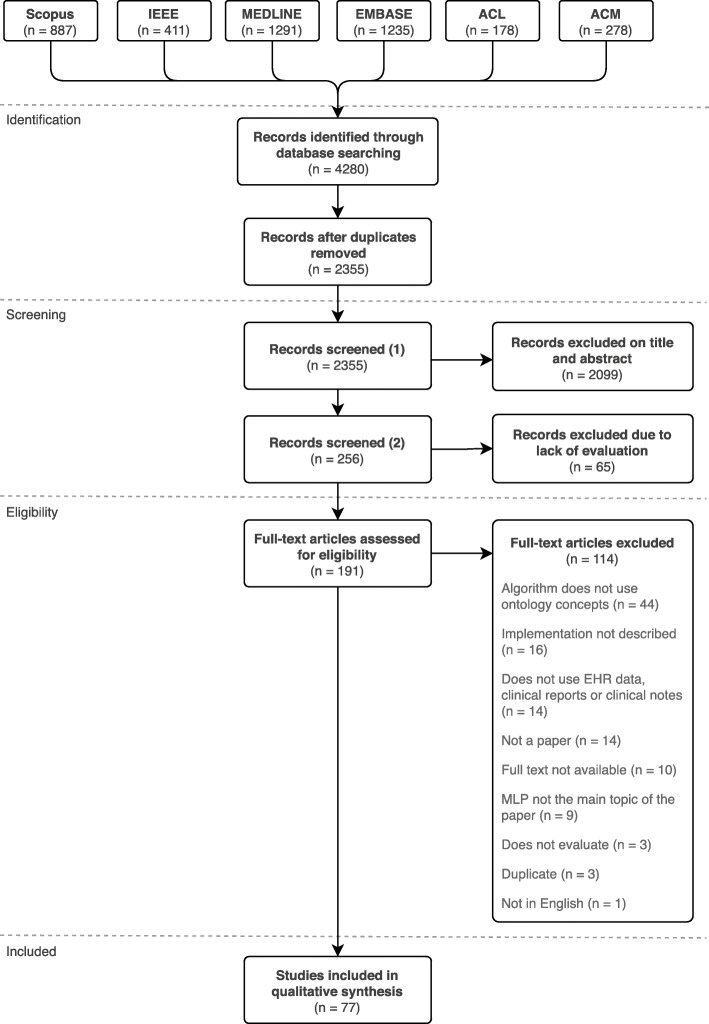
PRISMA flow diagram

The literature search generated a total of 2355 unique publications. After reviewing the titles and abstracts, we selected 256 publications for additional screening. Out of the 256 publications, we excluded 65 publications, as the described Natural Language Processing algorithms in those publications were not evaluated. The full text of the remaining 191 publications was assessed and 114 publications did not meet our criteria, of which 3 publications in which the algorithm was not evaluated, resulting in 77 included articles describing 77 studies. Reference checking did not provide any additional publications. The PRISMA flow diagram is presented in Fig. 1.

The induction process resulted in eight categories and ten associated NLP tasks that describe the objectives of the papers: computer-assisted coding, information comparison, information enrichment, information extraction, prediction, software development and evaluation, and text processing. Our definitions of these NLP tasks and the associated categories are given in Table 1 and Table 2.

**Table 1:** Induced objective tasks with their definition and an example

Induced NLP task(s)	Description	Example
**Concept detection** ^**1**^	Assign ontology concepts to phrases in free text (i.e., entity linking or annotation)	“Systolic blood pressure” can be represented as SNOMED-CT concept *271649006 | Systolic blood pressure (observable entity) |*
**Event detection**	Detect events in free text	“Patient visited the outpatient clinic in January 2020” is an event of type *Visit*.
**Relationship detection**	Detect semantic relationships between concepts in free text	The concept *Lung cancer* in “This patient was diagnosed with recurrent lung cancer” is related to the concept *Recurrence*.
**Text normalization**	Transform free text into a single canonical form	“This patient was diagnosed with influenza last year.” becomes “This patient be diagnose with influenza last year.”
**Text summarization**	Create a short summary of free text and possible restructure the text based on this summary	“Last year, this patient visited the clinic and was diagnosed with diabetes mellitus type 2, and in addition to his diabetes, the patient was also diagnosed with hypertension” becomes “Last year, this patient was diagnosed with diabetes mellitus type 2 and hypertension”.
**Classification**	Assign categories to free text	A report containing the text “This patient is not diagnosed yet” will be assigned to the category *Undiagnosed*.
**Prediction**	Create a predictive model based on free text	Predict the outcome of the APACHE score based on the (free-text) content in a patient chart.
**Identification**	Identify documents (e.g., reports or patient charts) that match a specific condition based on the contents of the document	Find all patient charts that describe patients with hypertension and a BMI above 30.
**Software development**	Develop new or build upon existing NLP software	A new algorithm was developed to map ontology concepts to free text in clinical reports.
**Software evaluation**	Evaluate the effectiveness of NLP software	The mapping algorithm has an F-score of 0.874.

^1.^Also known as Medical Entity Linking and Medical Concept Normalization

**Table 2:** Induced objective categories with their definition and associated NLP task(s)

Induced category	Induced NLP task(s)	Definition
**Computer-assisted coding**	Concept detection	Perform semi-automated annotation (i.e., with a human in the loop)
**Information comparison**	Concept detection Event detection Relationship detection	Compare extracted structured information to information available in free-text form
**Information enrichment**	Concept detection Event detection Relationship detection Text normalization Text summarization	Extract structured information from free text and attach this new information to the source
**Information extraction**	Concept detection Event detection Relationship detection	Extract structured information from free text
**Prediction**	Classification Prediction Identification	Use structured information to classify free-text reports, predict outcomes, or identify cases
**Software development** **and evaluation**	Software development Software evaluation	Develop new NLP software or evaluate new or existing NLP software
**Text processing**	Text normalization Text summarization	Transform free text into a new, more comprehensible form

Table 3 lists the included publications with their first author, year, title, and country. Table 4 lists the included publications with their evaluation methodologies. The non-induced data, including data regarding the sizes of the datasets used in the studies, can be found as supplementary material attached to this paper.

**Table 3:** Included publications and their first author, year, title, and country

Author	Year	Country	Challenge	Induced objective	Data origin	Dataset	Data language	Used system	Term. Sys.	In use	Source code	Ref
Afshar	2019	USA	No	Information extraction	Clinical Data Warehouse Data	Own	English	New (+ existing)	UMLS (CPT, HCPCS, ICD-10, ICD10CM / ICD9CM, LOINC, MeSH, SNOMED-CT, RxNorm)	Not listed	No, only links to cTAKES source code	[29]
Alnazzawi	2016	UK	No	Information enrichment	PhenoCHF corpus ^1^	Existing	English	Existing	UMLS	Not listed	Not applicable	[30]
Atutxa	2018	Spain	No	Information enrichment	EHR documents	Own	Spanish	New	ICD (SNOMED-CT for normalization)	Not yet, aim to embed it in human-supervised loop	Not listed	[31]
Barrett	2013	USA	No	Information extraction	Palliative care consult letters	Own	English	New	SNOMED CT	Not listed	No, but planned	[32]
Becker	2016	Germany	No	Information extraction	ShARe/CLEF corpus (2013) ^2^	Existing	German	Existing	SNOMED CT (English), UMLS (German)	Not yet, still under development	Not applicable	[33]
Becker	2019	Germany	No	Information extraction	Clinical notes of patients with known colorectal cancer	Own	German	New (+ existing)	UMLS	Yes, led to improved quality of care for colorectal patients	Not listed	[34]
Bejan	2015	USA	No	Information extraction	Discharge summaries and i2b2/VA challenge dataset (2010) ^3^	Own + Existing	English	Existing	UMLS	No	Not applicable	[35]
Castro	2010	Spain	No	Information extraction	Clinical notes with ‘most relevant information’	Own	Spanish	Existing	SNOMED CT	Not listed	Not applicable	[36]
Catling	2018	UK	No	Software development and evaluation	MIMIC-III dataset ^4^	Existing	English	New	ICD-9-CM	Not listed	Not listed	[37]
Chapman	2004	USA	No	Information extraction	Emergency department reports	Own	English	Existing	UMLS	Not listed	Not applicable	[38]
Chen	2016	USA	No	Information enrichment	Discharge summaries and progress notes	Own	English	New (+ existing)	UMLS	Not listed	Not listed	[39]
Chiaramello	2016	Italy	No	Information extraction	Clinical notes (cardiology, diabetology, hepatology, nephrology, and oncology)	Own	Italian	Existing	UMLS	Not listed	Not applicable	[40]
Chodey	2016	USA	SemEval (2014)	Information extraction	ICU Data: Discharge summaries, ECG, echo, and radiology	Existing	English	New (+ existing)	UMLS	Not listed	Not listed	[41]
Chung	2005	USA	No	Information extraction	Echocardiogram reports	Own	English	New (+ existing)	UMLS	Not yet, it will be used to populate a registry	Not listed	[42]
Combi	2018	Italy	No	Information extraction	VigiSegn (adverse drug reactions) reports	Own	Italian + English	New	MedDRA	Yes, implemented in VigiFarmaco	Pseudocode	[43]
De Bruijn	2011	Canada	i2b2/VA (2010)	Information extraction	Hospital discharge summaries and progress reports	Existing	English	New (+ existing)	UMLS	Not listed	Not listed	[44]
Deisseroth	2019	USA	No	Information extraction	Six sets of real patient data from four different medical centers.	Own	English	New	HPO	Not listed	Yes	[45]
Demner-Fushman	2017	USA	No	Software development and evaluation	BioScope ^5^, NCBI disease corpus ^6^, i2b2/VA challenge corpus (2010) ^3^, ShARe corpus ^7^, LHC test collection (biological/clinical journal abstracts)	Existing	English	New (+ existing)	UMLS	Yes, used in other papers identified in literature search	Yes	[46]
Divita	2014	USA	Parts: i2b2/VA (2010)	Software development and evaluation	Randomly selected clinical records from the most frequent document types	Own	English	New	UMLS (level 0 + 9)	Yes, used by VA Informatics and Computing Infrastructure	Yes	[47]
Duarte	2018	Portugal	No	Information enrichment	Death certificates, clinical bulletins, and autopsy reports	Own	Portuguese	New	ICD-10	Yes, used by Portugese Ministry of Health for near real-time death cause surveillance	Not listed	[48]
Falis	2019	UK	No	Information extraction	MIMIC-III dataset ^4^	Existing	English	New	ICD-9	Not listed	Not listed	[49]
Ferrão	2013	Portugal	No	Information enrichment	Inpatient adult episodes from the EHR	Own	Portuguese	New	ICD-9-CM	Not listed	Not listed	[50]
Gerbier	2011	France	No	Information extraction	Computerized emergency department medical records	Own	French	New	ICD-10, CCAM, SNOMED CT, ATC, MeSH, ICPC-2, DCR	Not yet, will be integrated into a CDSS	Not listed	[51]
Goicoechea Salazar	2013	Spain	No	Information enrichment	Diagnostic text from patient records	Own	Spanish	New	ICD-9-CM	Not listed	Not listed	[52]
Hamid	2013	USA	No	Classification	Notes of Iraq and Afghanistan veterans from the VA national clinical database	Own	English	Existing	UMLS	Not listed	Not applicable	[53]
Hassanzadeh	2016	Australia	No	Information extraction	ShARe/CLEF corpus (2013) ^2^	Existing	English	Existing	UMLS, SNOMED CT	Not applicable	Not applicable	[54]
Helwe	2017	Lebanon	No	Computer-assisted coding	MIMIC-III dataset	Existing	English	New	UMLS, ICD	Not listed	Not listed	[55]
Hersh	2001	USA	No	Information enrichment	Radiology image reports	Own	English	Existing	UMLS	No, still in development/testing	Pseudocode	[56]
Hoogendoorn	2015	Netherlands	No	Prediction	Consultation notes of patients in a primary care setting	Own	Dutch	New	SNOMED-CT, UMLS, ICPC	Not listed	Not listed	[57]
Jindal	2013	USA	i2b2 (2012)	Information extraction	i2b2 challenge corpus (2012) ^8^	Existing	English	New (+ existing)	UMLS, SNOMED CT, MeSH	Not listed	Not listed	[58]
Kang	2009	Korea	No	Information extraction	Discharge summaries	Own	Korean	New	KOMET, UMLS	Not listed	Not listed	[59]
Kersloot	2019	Netherlands	No	Information extraction	(Non-small cell) Lung cancer charts	Own	English	New (+ existing)	SNOMED CT	Not listed	Yes	[60]
König	2019	Germany	No	Software development and evaluation	Discharge letters from BASE-II study	Own	German	New (+ existing)	Wingert-Nomenclature	No, still has to prove its value	Not listed	[61]
Li	2015	USA	No	Information comparison	Clinical notes and discharge prescription lists	Own	English	New (+ existing)	UMLS, SNOMED CT, RxNorm	Not yet, plans to move to production	Pseudocode	[62]
Li	2019	USA	No	Information extraction	EHR notes	Own	English	New (+ existing)	UMLS, SNOMED CT, MedDRA	Not listed	Not listed	[63]
Lingren	2016	USA	No	Classification	Structured and unstructured data from two EHR databases	Own	English	New (+ existing)	UMLS, ICD-9, RxNorm	Not listed	Not listed	[12]
Liu	2019	USA	No	Information extraction	Clinical notes from different institutions + PubMed Case report abstracts	Own + Existing	English	Existing	HPO	Not listed	Not applicable	[64]
Lowe	2009	USA	No	Information extraction	Single-specimen pathology reports	Own	English	Existing	UMLS, SNOMED CT	Not listed	Not applicable	[65]
Luo	2014	USA	No	Information extraction	Pathology reports	Own	English	New (+ existing)	UMLS, SNOMED CT	Yes, currently working on project in multiple hospitals	Not listed	[66]
Meystre	2006	USA	No	Information enrichment	Clinical documents form adult inpatients in a cardiovascular unit	Own	English	New (+ existing)	UMLS (level 0), SNOMED CT	Not yet, testing in practice	Not listed	[67]
Meystre	2010	USA	i2b2 (2009)	Information extraction	i2b2 challenge dataset (2009) ^9^	Existing	English	New	UMLS	Not yet, possible integration in research infrastructure	Not listed	[68]
Minard	2011	France	i2b2/VA (2010)	Information extraction	i2b2/VA challenge corpus (2010) ^3^	Existing	English	New (+ existing)	UMLS	Not listed	Not listed	[69]
Mishra	2019	USA	No	Information extraction	Clinical notes from NIH Clinical Center data warehouse	Own	English	Existing	UMLS, HPO	Not listed	Not applicable	[70]
Nguyen	2018	Australia	No	Computer-assisted coding	Hospital progress notes	Own	English	New (+ existing)	SNOMED CT, ICD-10-AM	Not listed	Not listed	[71]
Oellrich	2015	UK	No	Information extraction	PubMed abstracts, clinical trial information, i2b2/VA challenge corpus (2010) ^3^, SHARE/CLEF (2013) ^2^	Existing	English	Existing	UMLS	Not listed	Not applicable	[72]
Patrick	2011	Australia	i2b2/VA (2010)	Information extraction	i2b2/VA challenge corpus (2010) ^3^	Existing	English	New	UMLS, SNOMED CT	Not listed	Not listed	[73]
Pérez	2018	Spain	No	Text processing	Spontaneous DTs randomly selected entries	Own	Spanish	New	ICD	Not listed	Not listed	[74]
Reátegui	2018	Canada	No	Information extraction	i2b2 challenge corpus (2008) ^10^	Existing	English	New (+ existing)	UMLS, SNOMED CT, RxNorm	Not listed	Not listed	[75]
Roberts	2011	USA	i2b2/VA (2010)	Information extraction	i2b2/VA challenge corpus (2010) ^3^	Existing	English	New (+ existing)	UMLS, ICD-9	Not listed	Not listed	[76]
Rousseau	2019	USA	No	Information comparison	ED encounters for patients with headaches who received head CT	Own	English	Existing	UMLS: SNOMED CT, RadLex	Not listed	Not applicable	[77]
Savova	2010	USA	i2b2 (2006, 2008)	Information extraction	Subset of clinical notes from the EMR	Own	English	New (+ existing)	UMLS, SNOMED CT, RxNorm	Yes, used in other papers identified in literature search	Yes	[78]
Shivade	2015	USA	i2b2/UTHealth (2014)	Classification	i2b2 challenge corpus (2014) ^11^	Existing	English	Existing	UMLS	Not listed	Not applicable	[11]
Shoenbill	2019	USA	No	Information extraction	EHR notes from hypertension patients	Own	English	Existing	UMLS, SNOMED CT	Not listed	Not applicable	[79]
Sohn	2014	USA	No	Information extraction	Clinical notes with medication mentions	Own	English	New	RxNorm	Not listed	Yes	[80]
Solti	2008	USA	No	Information enrichment	Cardiology ambulatory progress notes	Own	English	Existing	UMLS	Not listed	Not applicable	[81]
Soriano	2019	Spain	No	Information extraction	clinical emergency discharge reports	Own	Spanish	New	SNOMED CT	Not yet	Yes	[82]
Soysal	2018	USA	Parts: i2b2 (2009 + 2010), ShARe/CLEF (2013), Sem-EVAL (2014)	Software development and evaluation	Discharge summaries from the i2b2/VA challenge corpus (2010) ^3^, outpatient clinic visit notes, mock clinical documents	Own + Existing	English	New	UMLS	Yes, used by various institutions and industrial entities	Yes	[83]
Spasić	2015	UK	No	Information extraction	MRI reports of patients	Own	English	New (+ existing)	TRAK, UMLS, MEDCIN, RadLex	Not listed	Yes	[84]
Strauss	2013	USA	No	Information extraction	Pathology reports of breast and prostate cancer patients	Own	English	New	SNOMED CT	Not listed	Yes	[85]
Sung	2018	Taiwan	No	Information extraction	Cases of adult patients with AIS	Own	English	Existing	UMLS	Not listed	Not applicable	[86]
Tchechmedjiev	2018	France	No	Information extraction	Quaero (French MEDLINE abstract titles + EMEA drug labels) + CépiDC (ICD-10 coding of death certificates)	Existing	French	New (+ existing)	UMLS terminologies (ICD-10)	Yes, available in SIFR BioPortal	Yes	[87]
Ternois	2018	France	No	Classification	Endoscopy reports written between 2015 and 2016	Own	French	New	CCAM	Not listed	Not listed	[88]
Travers	2004	USA	No	Information extraction	Chief complaint text entries for all emergency department visits	Own	English	New	UMLS	Not listed	Not listed	[89]
Tulkens	2019	Belgium	No	Information extraction	i2b2/VA challenge corpus (2010) ^3^	Existing	English	New (+ existing)	UMLS	Not listed	Yes	[90]
Usui	2018	Japan	No	Prediction	Electronic medication history data from pharmacy	Own	Japanese	New	ICD-10	Not yet, expect to use it	Not listed	[91]
Valtchinov	2019	USA	No	Classification	Radiology reports, emergency department notes + other clinical reports	Own	English	Existing	SNOMED CT, RadLex	Not listed	Not applicable	[92]
Wadia	2018	USA	No	Classification	Chest CT reports	Own	English	Existing	SNOMED CT, UMLS	Not listed	Not applicable	[93]
Walker	2019	USA	No	Information extraction	Treatment sites from EMR	Own	English	New	UMLS	Not listed	Not listed	[94]
Xie	2019	China	No	Information extraction	MIMIC-III dataset ^4^	Existing	English	New	ICD-9-CM, ICD-10	Not listed	Not listed	[95]
Xu	2011	USA	No	Classification	CRC patient cases from the Synthetic Derivative database	Own	English	Existing	UMLS	No, still under development	Not applicable	[96]
Yadav	2013	USA	No	Prediction	Emergency department CT imaging reports	Own	English	Existing	UMLS	Not listed	Yes, command line command	[97]
Yao	2019	USA	No	Prediction	i2b2 challenge corpus (2008) ^10^	Existing	English	New (+ existing)	UMLS	Not listed	Part (Sorl)	[98]
Zeng	2018	USA	No	Classification	Progress notes and breast cancer surgical pathology reports	Own	English	New (+ existing)	UMLS	Not listed	Not listed	[99]
Zhang	2013	USA	No	Information extraction	i2b2/VA challenge corpus (2010) ^3^ and GENIA corpus (MEDLINE abstracts)	Existing	English	New	UMLS	Not listed	Not listed	[100]
Zhou	2006	USA	No	Information extraction	Records of patients with breast complaints	Own	English	New	UMLS	No, still under development	Not listed	[101]
Zhou	2011	USA	No	Software development and evaluation	COPD and CAD patients	Own	English	New	SNOMED CT, RxNorm, UMLS, PPL, MDD, HL7 value sets	Yes, described in other paper (103])	Not listed	[102]
Zhou	2014	USA	No	Information extraction	Admission notes and discharge summaries	Own	English	Existing	SNOMED CT, HL7 RoleCodes	Not listed	Not applicable	[103]

1. PhenoCHF corpus: narrative reports from electronic health records (EHRs) and literature articles

2. ShARe/CLEF corpus (2013): narrative clinical reports

3. i2b2/VA challenge dataset (2010): discharge summaries and progress reports

4. MIMIC-III dataset: demographics, vital sign measurements, laboratory test results, procedures, medications, caregiver notes, imaging reports, and mortality

5. BioScope corpus: medical free texts, biological full papers and biological scientific abstracts

6. NCBI disease corpus: PubMed abstracts

7. ShARe corpus: deidentified clinical free-text notes from the MIMIC II database

8. i2b2 challenge corpus (2012): discharge summaries

9. i2b2 challenge dataset (2009): de-identified hospital discharge summaries

10. i2b2 challenge corpus (2008): discharge summaries of overweight and diabetic patients

11. i2b2 challenge corpus (2014): longitudinally ordered clinical notes from three cohorts of diabetic patients

**Table 4:** Included publications and their evaluation methodologies

Author	Year	Ref. std.	Validation	External	Generalizability ^**a**^	Ref
Afshar	2019	Existing EHR data	Hold-out validation (train, test, development)	No	No, validation is needed	[29]
Alnazzawi	2016	Existing annotated corpus	External	ShARe/CLEF, NCBI disease, Heart failure and pulmonary embolism corpora	Yes, achieves competitive performance on other corpora	[30]
Atutxa	2018	Manual retrospective review	Hold-out validation (train, test, development)	No	Yes, easily portable to other languages	[31]
Barrett	2013	Manual annotations	10-fold cross validation	Multiple datasets (different provider)	Yes, expect that it is generalizable	[32]
Becker	2016	Existing annotated corpus	Not used	No	Not listed	[33]
Becker	2019	Manual annotations	Hold-out validation (train, test, development)	No	Not listed	[34]
Bejan	2015	Manual annotations	External	i2b2 data (2010)	Yes, good performance on the i2b2 dataset, even though not optimized on it	[35]
Castro	2010	Manual annotations	Not used	No	Not listed	[36]
Catling	2018	Existing annotated corpus	Hold-out validation (train, test, development)	No	Not listed	[37]
Chapman	2004	Manual annotations	Not used	No	Yes, generalizable to other domains within and outside of bio surveillance	[38]
Chen	2016	Manual annotations	10-fold cross validation	No	Not listed	[39]
Chiaramello	2016	Manual annotations	Not used	No	Not listed	[40]
Chodey	2016	Existing annotated corpus	Hold-out validation (train, test)	No	Not listed	[41]
Chung	2005	Manual annotations	Hold-out validation (train, test)	Reports from a second hospital	Not listed	[42]
Combi	2018	Manual annotations	Not used	No	Not listed	[43]
deBruijn	2011	Existing annotated corpus	15-fold cross validation	No	Not listed	[44]
Deisseroth	2019	Manual annotations	Hold-out validation (train, test)	Data from a second hospital	Yes, it can be immediately incorporated into clinical practice	[45]
Demner-Fushman	2017	Existing annotated corpus	External	Multiple datasets	Not listed	[46]
Divita	2014	Manual annotations	Not used	No	Not listed	[47]
Duarte	2018	Manual annotations	Hold-out validation (train, test)	Second dataset	Not listed	[48]
Falis	2019	Existing annotated corpus	Hold-out validation (train, test, development)	No	Yes, method is not specific to an ontology, and could be used for a graph of any formation	[49]
Ferrão	2013	Existing EHR data	Hold-out validation (train, test)	No	Not listed	[50]
Gerbier	2011	Manual annotations	Hold-out validation (train, test)	No	Yes, it could also serve other types of clinical decision support systems	[51]
Goicoechea Salazar	2013	Manual annotations	Hold-out validation (train, test)	No	Not listed	[52]
Hamid	2013	Manual annotations	10-fold cross validation	No	Possible, the classifier may be applicable in academic hospital samples	[53]
Hassanzadeh	2016	Existing annotated corpus	Hold-out validation (train, test)	No	Not applicable	[54]
Helwe	2017	Existing annotated corpus	Hold-out validation (train, test, development)	No	Not listed	[55]
Hersh	2001	Manual annotations	Hold-out validation (train, test)	No	Not listed	[56]
Hoogendoorn	2015	Existing EHR data	5-fold cross validation	No	Not listed	[57]
Jindal	2013	Existing annotated corpus	Hold-out validation (train, test)	No	Yes, broad applicability	[58]
Kang	2009	Manual annotations	Hold-out validation (train, test)	No	Yes, extensible to other languages	[59]
Kersloot	2019	Manual annotations	Hold-out validation (development, test)	No	Possible, but external validation is needed	[60]
König	2019	Existing EHR data	Not used	No	Still to be tested	[61]
Li	2015	Manual annotations	10-fold cross validation	No	Not listed	[62]
Li	2019	Existing annotated corpus	Hold-out validation (train, test, development)	No	Not listed	[63]
Lingren	2016	Manual annotations	Hold-out validation (train, test, development)	No	Not listed	[12]
Liu	2019	Manual annotations	Not used	No (but multiple datasets / non-trained)	No, limited because of NYP/CUIMC and Mayo notes.	[64]
Lowe	2009	Manual retrospective review	Hold-out validation (train, test)	No	Yes, has the potential to index other classes of clinical documents	[65]
Luo	2014	Existing EHR data	10-fold cross validation	No	No, challenging, not currently working on it	[66]
Meystre	2006	Manual retrospective review	Not used	No	Not listed	[67]
Meystre	2010	Existing annotated corpus	Hold-out validation (train, test)	No	Not listed	[68]
Minard	2011	Existing annotated corpus	Hold-out validation (train, test, development)	No	Not listed	[69]
Mishra	2019	Manual annotations	Not used	No	Not listed	[70]
Nguyen	2018	Existing EHR data	Not listed	No	Not listed	[71]
Oellrich	2015	Existing annotated corpus	External	Multiple datasets	Not listed	[72]
Patrick	2011	Existing annotated corpus	10-fold cross validation	No	Yes, adaptable to different requirements in clinical information extraction and classification by choosing relevant feature sets	[73]
Pérez	2018	Existing annotated corpus	Hold-out validation (train, test, development)	No	Yes, extensible to different hospital-sections and hospitals	[74]
Reátegui	2018	Existing annotated corpus	Not used	No	Not listed	[75]
Roberts	2011	Existing annotated corpus	Hold-out validation (train, test)	No	Not listed	[76]
Rousseau	2019	Manual annotations	Not used	No	Not listed	[77]
Savova	2010	Manual annotations	10-fold cross validation	No	Yes, implemented in several applications	[78]
Shivade	2015	Manual annotations	Hold-out validation (train, test)	No	Not listed	[11]
Shoenbill	2019	Manual annotations	Hold-out validation (train, test)	No	Yes, can allow further evaluation and improvement in care delivery models and treatment approaches to multiple chronic illnesses	[79]
Sohn	2014	Manual annotations	Hold-out validation (train, test, development)	No	Yes, with adaptions: create flexible mechanism for adaptation process	[80]
Solti	2008	Manual annotations	Hold-out validation (train, test)	No	Not listed	[81]
Soriano	2019	Manual annotations	Not listed	No	Not listed	[82]
Soysal	2018	Existing annotated corpus	Hold-out validation (train, test)	No	Yes, can be used to quickly develop customized clinical information extraction pipelines	[83]
Spasić	2015	Manual annotations	Hold-out validation (train, test)	No	Not listed	[84]
Strauss	2013	Manual annotations	Not used	No	Yes, can be shared between institutions and used to support clinical + epidemiological research	[85]
Sung	2018	Manual annotations	Not listed	No	Not listed	[86]
Tchechmedjiev	2018	Existing annotated corpus	Hold-out validation (train, test, development)	No	Yes, but not universally	[87]
Ternois	2018	Existing EHR data	5-fold cross validation + Hold-out validation (train, test)	No	Not listed	[88]
Travers	2004	Manual retrospective review	Not used	No	Not listed	[89]
Tulkens	2019	Existing annotated corpus	Hold-out validation (train, test, development)	No	Not listed	[90]
Usui	2018	Manual annotations	Not used	No	Not listed	[91]
Valtchinov	2019	Manual annotations	Not used	No	No	[92]
Wadia	2018	Manual annotations	Not used	No	Not listed	[93]
Walker	2019	Manual retrospective review	Hold-out validation (development, test)	No	Yes, it can be incorporated in institutional data warehouse	[94]
Xie	2019	Existing annotated corpus	Hold-out validation (train, test, development)	No	Not listed	[95]
Xu	2011	Manual annotations	Hold-out validation (train, test)	No	Yes, generable approach to combine information from heterogeneous data sources in EHRs	[96]
Yadav	2013	Manual annotations	Not used	*No*	Yes, should be broadly applicate to outcomes of clinical interest	[97]
Yao	2019	Existing annotated corpus	Hold-out validation (train, test)	No	Not listed	[98]
Zeng	2018	Manual annotations	5-fold cross validation + Hold-out validation (train, test)	No	Yes, potential to be replicated	[99]
Zhang	2013	Existing annotated corpus	External	Two different sets with same settings	Yes, can be adapted to different semantic categories and text genres	[100]
Zhou	2006	Manual annotations	5-fold cross validation	No	Not listed	[101]
Zhou	2011	Manual retrospective review	Hold-out validation (train, test)	No	Not listed	[102]
Zhou	2014	Manual annotations	Not used	No	Not listed	[103]

^a^ As reported by authors

Table 5 summarizes the general characteristics of the included studies and Table 6 summarizes the evaluation methods used in these studies. In all 77 papers, we found twenty different performance measures (Table 7).

**Table 5:** Characteristics of the included studies

Description	n (%)	References
**Main objective**		
Information extraction	45 (58%)	[29, 32–36, 38, 40–45, 49, 51, 58–60, 63–66, 68–70, 72, 73, 75, 76, 78–80, 82, 84–87, 89, 90, 94, 95, 100, 101, 103, 104]
Information enrichment	9 (12%)	[30, 31, 39, 48, 50, 52, 56, 67, 81]
Classification	8 (10%)	[11, 12, 53, 88, 92, 93, 96, 99]
Software development and evaluation	6 (7.8%)	[37, 46, 47, 61, 83, 102]
Prediction	4 (5.2%)	[57, 91, 97, 98]
Information comparison	2 (2.6%)	[62, 77]
Computer-assisted coding	2 (2.6%)	[55, 71]
Text processing	1 (1.3%)	[74]
**Part of challenge**		
i2b2 (Informatics for Integrating Biology and the Bedside)	10 (13%)	[11, 44, 47, 58, 68, 69, 73, 76, 78, 83]
Entire system	8 (10%)	[11, 44, 58, 68, 69, 73, 76, 78]
Parts of the system	2 (2.6%)	[47, 83]
SemEval (Semantic Evaluation)	2 (2.6%)	[41, 83]
Entire system	1 (1.3%)	[41]
Parts of the system	1 (1.3%)	[83]
ShARe/CLEF (Shared Annotated Resources/Conference and Labs of the Evaluation Forum)	1 (1.3%)	[83]
Parts of the system	1 (1.3%)	[83]
**Dataset: language**		
English	60 (78%)	[11, 12, 29, 30, 32, 35, 37–39, 41–47, 49, 53, 55, 56, 58, 60, 62–73, 75–81, 83–86, 89, 90, 92–104]
Spanish	5 (6.5%)	[31, 36, 52, 74, 82]
French	3 (3.9%)	[51, 87, 88]
German	3 (3.9%)	[33, 34, 61]
Italian	2 (2.6%)	[40, 43]
Portuguese	2 (2.6%)	[48, 50]
Dutch	1 (1.3%)	[57]
Japanese	1 (1.3%)	[91]
Korean	1 (1.3%)	[59]
**Dataset: Origin**		
Data present in institute	55 (71%)	[12, 29, 31, 32, 34–36, 38–40, 42, 43, 45, 47, 48, 50–53, 56, 57, 59–67, 70, 71, 74, 77–86, 88, 89, 91–94, 96, 97, 99, 101–103]
Existing dataset	25 (33%)	[11, 30, 33, 35, 37, 41, 44, 46, 49, 55, 58, 64, 68, 69, 72, 73, 75, 76, 83, 87, 90, 95, 98, 100, 104]
Included reference to dataset	21 (27%)	[11, 30, 35, 37, 41, 44, 46, 49, 55, 58, 64, 72, 75, 76, 83, 87, 90, 95, 98, 100, 104]
**Training of algorithm**		
Trained	47 (61%)	[11, 12, 29, 31, 32, 34, 37, 39, 41, 42, 44, 45, 48–53, 55–59, 62, 63, 65, 66, 68, 69, 73, 74, 76, 78–84, 87, 88, 90, 95, 96, 98, 99, 104]
Not listed	3 (3.9%)	[30, 101, 102]
**Development of algorithm**		
Use of development set	16 (21%)	[12, 29, 31, 34, 37, 49, 55, 60, 63, 69, 74, 80, 87, 90, 94, 95]
Not listed	4 (5.2%)	[30, 82, 83, 101]
**Used NLP system or algorithm**		
New NLP system or algorithm	29 (38%)	[31, 32, 37, 43, 45, 47–52, 55, 57, 59, 68, 73, 74, 80, 82, 83, 85, 88, 89, 91, 94, 95, 100–102]
New NLP system or algorithm with existing components	25 (33%)	[12, 29, 34, 39, 41, 42, 44, 46, 58, 60–63, 66, 67, 69, 71, 75, 76, 78, 84, 87, 90, 98, 99]
Existing NLP system or algorithm	23 (30%)	[11, 30, 33, 35, 36, 38, 40, 53, 56, 64, 65, 70, 72, 77, 79, 81, 86, 93, 96, 97, 103, 104]
**Use in practice**		
Plans to implement / still under development and testing	12 (16%)	[31, 33, 51, 56, 62, 66–68, 82, 91, 96, 101]
Implemented in practice	10 (13%)	[34, 42, 43, 46–48, 78, 83, 87, 102]
**Availability of code**		
Published algorithm or source code	15 (20%)	[31, 45–47, 60, 78, 80, 82–85, 87, 90, 97, 98]
Pseudocode in manuscript	3 (3.9%)	[43, 56, 62]
Planning to publish algorithm or source code	1 (1.3%)	[32]
Not applicable, used an existing system	20 (26%)	[11, 30, 33, 35, 36, 38, 40, 53, 64, 65, 70, 72, 77, 79, 81, 86, 93, 96, 103, 104]

**Table 6:** Evaluation methods of the included studies

Description	n (%)	References
**Evaluation: Reference standard**		
Manual annotations	40 (52%)	[11, 12, 32, 34–36, 38–40, 42, 43, 45, 47, 48, 51–53, 56, 59, 60, 62, 64, 70, 77–82, 84–86, 91–93, 96, 97, 99, 101, 103]
Existing annotated corpus	24 (31%)	[30, 33, 37, 41, 44, 46, 49, 55, 58, 63, 68, 69, 72–76, 83, 87, 90, 95, 98, 100, 104]
Existing EHR data	7 (9.1%)	[29, 50, 57, 61, 66, 71, 88]
Manual retrospective review	6 (7.8%)	[31, 65, 67, 89, 94, 102]
**Evaluation: Validation**		
Hold-out validation	40 (52%)	[11, 12, 29, 31, 34, 37, 41, 42, 45, 48–52, 55, 56, 58–60, 63, 65, 68, 69, 74, 76, 79–81, 83, 84, 87, 88, 90, 94–96, 98, 99, 102, 104]
Cross-validation	12 (16%)	[32, 39, 44, 53, 57, 62, 66, 73, 78, 88, 99, 101]
External validation	9 (12%)	[30, 32, 35, 42, 45, 46, 48, 72, 100]
Solely external validation	5 (6.5%)	[30, 35, 46, 72, 100]
In addition to another type of validation	4 (5.2%)	[32, 42, 45, 48]
Not performed or not listed	22 (29%)	[33, 36, 38, 40, 43, 47, 61, 64, 67, 70, 71, 75, 77, 82, 85, 86, 89, 91–93, 97, 103]
**Generalizability**		
Claimed	23 (30%)	[30–32, 35, 38, 45, 49, 51, 58, 59, 65, 73, 74, 78–80, 83, 85, 87, 94, 96, 97, 100]
Externally validated	5 (6.5%)	[30, 32, 35, 45, 100]
**Comparison**		
Compared to other existing algorithms or models	24 (31%)	[30, 35, 39, 45–47, 49, 58, 60, 63, 64, 72, 75, 80, 83, 87, 90, 94, 95, 98–101, 104]
Tested difference in outcomes for statistical significance	4 (5.2%)	[35, 39, 60, 63]

**Table 7:** Performance measures used in the included studies

Description	Formula	n (%)	References
Confusion Matrix	Lists the True Positives (TP), True Negatives (TN), False Positives (FP), False Negatives (FN), and the Total (n) amount in a 2 × 2 contingency Table. TP: Text annotated with ontology concept when ontology concept is present in reference standard TN: Text not annotated with ontology concept when ontology concept is absent in reference standard FP: Text annotated with ontology concept when ontology concept is absent in reference standard FN: Text not annotated with ontology concept when ontology concept is present in reference standard	12 (16%)	[34, 44, 47, 51, 56, 58, 60, 61, 84, 87, 91, 93]
**Performance measures**			
Recall	$\frac{TP}{FN+ TP}$	68 (88%)	[11, 12, 29–31, 33–53, 56–58, 60–64, 66–73, 75–88, 90–94, 96, 99–104]
Precision	$\frac{TP}{FP+ TP}$	66 (86%)	[11, 12, 29–31, 33–36, 38–51, 53, 56–58, 60–73, 75–88, 90, 91, 93, 94, 96, 99–104]
F-score	$2\bullet \frac{Precision\bullet Recall}{Precision+ Recall}$	57 (74%)	[11, 12, 30, 31, 33–36, 39–41, 44, 46–50, 52, 53, 55, 57–63, 66–73, 75–80, 82–84, 86–88, 90, 91, 95, 96, 98–100, 102–104]
Accuracy	$\frac{TP+ TN}{n}$	11 (14%)	[30, 32, 34, 41, 48, 52, 67, 74, 78, 92, 96]
Specificity	$\frac{TN}{FP+ TN}$	6 (7.8%)	[29, 34, 85, 92, 93, 96]
AUC	Not applicable	5 (6.5%)	[29, 39, 57, 95, 99]
Kappa	$\frac{p_o-\kern0.5em {p}_e}{1-{p}_e}=1-\frac{1-{p}_o}{1-{p}_e}$	3 (3.9%)	[85, 89, 97]
Processing time	Not applicable	3 (3.9%)	[32, 47, 83]
Negative Predictive Value	$\frac{TN}{FN+ TN}$	3 (3.9%)	[29, 85, 93]
False Positive Rate	$\frac{FP}{FP+ TN}$	1 (1.3%)	[34]
False Negative Rate	$\frac{FN}{TP+ FN}$	1 (1.3%)	[34]
Information entropy	$-{\sum}_{i=1}^n{P}_i\ \mathit{\log}\left({P}_i\right)$	1 (1.3%)	[64]
Mean Reciprocal Rank	$\frac{1}{Q}{\sum}_{i=1}^Q\frac{1}{{\mathit{\operatorname{rank}}}_i}$	1 (1.3%)	[74]
Initial annotator agreement	Not applicable	1 (1.3%)	[79]
Match/no match (%)	Not applicable	1 (1.3%)	[89]
Overgeneration	$\frac{FP}{TP+ FP}$	1 (1.3%)	[93]
Undergeneration	$\frac{FN}{TP+ FN}$	1 (1.3%)	[68]
Error	$\frac{FN+ FP}{TP+ FN+ FP}$	1 (1.3%)	[68]
Fallout	$\frac{FP}{TN+ FP}$	1 (1.3%)	[68]
Mean Standard Error	$\frac{1}{n}{\sum}_{i=1}^n{\left({Y}_i-{\hat{Y}}_i\right)}^2$	1 (1.3%)	[57]

## Discussion

In this systematic review, we reviewed the current state of NLP algorithms that map clinical text fragments onto ontology concepts with regard to their development and evaluation, in order to propose recommendations for future studies.

### Main findings and recommendations

We identified 256 studies that reported on the development of such algorithms, of which 68 did not evaluate the performance of the system. We included 77 studies. Many publications did not report their findings in a structured way, which made it challenging to extract all the data in a reliable manner. We discuss our findings and recommendations in the following five categories: Used NLP systems and algorithms, Used data, Evaluation and validation, Presentation of results, and Generalizability of results. A checklist for determining if the recommendations are followed in the reporting of an NLP study is added as supplementary material to this paper.

### Used NLP systems and algorithms

A variety of NLP systems are used in the reviewed studies. Researchers use existing systems (*n* = 29, 38%), develop new systems with existing components (*n* = 25, 33%), or develop a completely new system (*n* = 23, 30%). Most studies, however, do not publish their (adapted) source code (*n* = 57, 74%), and a description of the algorithm in the final publication is often not detailed enough to replicate it. To ensure reproducibility, implementation details, including details on data processing, and preferably the source code should be published, allowing other researchers to compare their implementations or to reproduce the results. Based on these findings, we formulated three recommendations (Table 8).

**Table 8:** Recommendation regarding the use of systems and algorithms

1. Describe the system or algorithm that is used or the system that is developed for the specific NLP task. 1. When an existing NLP system or algorithm is used, describe how it is set up, how it is implemented in practice, and if and how the implementation differs from the original implementation. 2. When a new system is developed, describe the components and features used in the system, and preferably include a flow chart that explains how these elements work together. 2. Include the source code of the developed algorithm as supplementary material to the publication or upload the source code to a repository such as GitHub. 3. Specify which ontologies are used in the encoding task, including the version of the ontology. 1. If a new ontology is developed for the encoding task, report on the development and content of the ontology and rationale for the development of a new ontology instead of the use of an existing one. The MIRO guidelines could be used to structure the report [105].	

### Used data

Most authors evaluate their algorithms with manual annotations (*n* = 40, 52%) and use data present in their institutions (*n* = 55, 71%). However, it is not clear what these datasets consist of. Most studies describe the data as ‘reports’, ‘notes’, or ‘summaries’, but do not list the contents or example rows from the dataset. It is, therefore, not clear what types of patients and what specific types of data are included, making the study hard to reproduce. Finally, we found a wide range of dataset sizes and formats. The training datasets, for example, ranged from 10 clinical notes to 636.439 discharge reports. The use of small datasets can result in an overfitted algorithm that either performs well on the dataset, but not on an external dataset, or performs poorly, for the algorithm was only trained on a specific type of data. More difficult recognition tasks require more data, and therefore sample size planning is recommended [106]. To improve the description and availability of datasets used in NLP studies, we formulated three recommendations (Table 9).

**Table 9:** Recommendation regarding the use of data

1. To ensure that new algorithms can be compared against your system, aim to publish the used training, development, and validation data in a data repository. 1. In case the data cannot be published, determine if the data can be accessed on request or can be used in a federated learning approach (i.e., a learning process in which the data owners collaboratively train a model in which process any data owner does not expose the data to others [107]). 2. In case a reference standard is used, include information about the origin of the data (external dataset, subset of the dataset) and the characteristics of the data in the dataset. If possible, reference the dataset using a DOI or URL. 3. If an external dataset is used, give a short description of the data present in the dataset and reference the source of the dataset.	

### Evaluation and validation

Evaluation of the algorithm determines its performance on the dataset, and validation determines if the algorithm is not overfitted on that dataset and thus if the algorithm might work on other datasets as well. Over one-fourth of the studies (*n* = 68, 27%) that we identified did not evaluate their algorithms. In addition, 22 included studies (29%) did not validate the developed algorithm. A statement claiming that an algorithm can be used in clinical practice can be questioned if the algorithm has not been evaluated and validated. Across all studies, 20 performance measures were used. To harmonize evaluation and validation efforts, we formulated three recommendations (Table 10).

**Table 10:** Recommendation regarding the evaluation and validation of Natural Language Processing algorithms

1. Perform an evaluation using generic (i.e., precision, recall, and F-score) performance measures and appropriate aspects of evaluation including discrimination, calibration, and preferably accuracies of predictions (e.g., AUC, calibration graphs, and the Brier score). 1. Include a motivation for the choice of measures, with references to existing literature where appropriate (e.g., Sokolova and Lapalme’s analysis of performance measures [108]). 2. Perform an error analysis and discuss the errors in the Discussion section of the paper. Include possible changes to the algorithm that could improve its performance for these specific errors. 3. When using a non-probabilistic NLP method: determine the cut-off value (a priori) for a ‘good’ test result before evaluating the algorithm. Elaborate why this cut-off value is chosen.	

### Presentation of results

Authors report the evaluation results in various formats. Only twelve articles (16%) included a confusion matrix which helps the reader understand the results and their impact. Not including the true positives, true negatives, false positives, and false negatives in the Results section of the publication, could lead to misinterpretation of the results of the publication’s readers. For example, a high F-score in an evaluation study does not directly mean that the algorithm performs well. There is also a possibility that out of 100 included cases in the study, there was only one true positive case, and 99 true negative cases, indicating that the author should have used a different dataset. Results should be clearly presented to the user, preferably in a table, as results only described in the text do not provide a proper overview of the evaluation outcomes (Table 11). This also helps the reader interpret results, as opposed to having to scan a free text paragraph. Most publications did not perform an error analysis, while this will help to understand the limitations of the algorithm and implies topics for future research.

**Table 11:** Recommendation regarding the presentation of results

1. Report the outcomes of the evaluation in a clear manner, preferably in a table accompanied by a textual description of the outcomes. 1. Aim to include a confusion matrix in the reporting of the outcomes. 2. Use figures if they contribute to the making the results more readable and understandable for the reader. If a figure is used, make sure that the data is also available in the text or in a table.	

### Generalizability of results

88% of the studies did not perform external validation (*n* = 68). Of the studies that claimed that their algorithm was generalizable, only 22% (*n* = 5) assessed this claim through external validation. However, one cannot claim generalizability without testing for it. Moreover, in 19% (*n* = 3) of the cases where external datasets were used, the datasets were not referenced and only listed in the text of the article, making it harder to find the used data and reproduce the results. Algorithm performance should be compared to that of other state-of-the-art algorithms, as this helps the reader decide whether the new algorithm could be considered useful for clinical practice. However, only 24 studies (31%) made this comparison, and four of those studies (17%) tested the performance difference for statistical significance. We also found that the authors’ descriptions of generalizability are rather ambiguous and unclear. We formulated five recommendations regarding the generalizability of results (Table 12).

**Table 12:** Recommendation regarding the generalizability of results

1. Compare the results of the evaluated algorithm with other algorithms by using the same dataset as reported in the publication of the other algorithm or by processing the same dataset with another algorithm available through the literature. Report the outcomes of both experiments and test for statistical significance. 2. Describe in what setting the research is performed. Include if the research is part of a challenge (e.g., i2b2 challenge), or that the research is carried out in a specific institute or department. 3. Before claiming generalizability, perform external validation by testing the algorithm on a different, external dataset from other research projects or other publicly available datasets. Aim to use a dataset with a different case mix, different individuals, and different types of text. 4. Determine and describe if there are potential sources of bias in data selection, data use by the NLP algorithm or system, and evaluation. 5. When claiming generalizability, clearly describe the conditions under which the algorithm can be used in a different setting. Describe for which population, domain, and type and language of data the algorithm can be used.	

### Strengths

Our study has three main strengths: First, to our knowledge, this is the first systematic review that focuses on the evaluation of NLP algorithms in medicine. Second, we used a large number of databases for our search, resulting in publications from many different sources, such as medical journals and computer science conferences. Third, we used existing statements and guidelines and harmonized them to induce our findings and used these findings to propose a list of recommendations.

### Limitations

Several limitations of our study should be noted as well. First, we only focused on algorithms that evaluated the outcomes of the developed algorithms. Second, the majority of the studies found by our literature search used NLP methods that are not considered to be state of the art. We found that only a small part of the included studies was using state-of-the-art NLP methods, such as word and graph embeddings. This indicates that these methods are not broadly applied yet for algorithms that map clinical text to ontology concepts in medicine and that future research into these methods is needed. Lastly, we did not focus on the outcomes of the evaluation, nor did we exclude publications that were of low methodological quality. However, we feel that NLP publications are too heterogeneous to compare and that including all types of evaluations, including those of lesser quality, gives a good overview of the state of the art.

## Conclusion

In this study, we found many heterogeneous approaches to the development and evaluation of NLP algorithms that map clinical text fragments to ontology concepts and the reporting of the evaluation results. Over one-fourth of the publications that report on the use of such NLP algorithms did not evaluate the developed or implemented algorithm. In addition, over one-fourth of the included studies did not perform a validation and nearly nine out of ten studies did not perform external validation. Of the studies that claimed that their algorithm was generalizable, only one-fifth tested this by external validation. Based on the assessment of the approaches and findings from the literature, we developed a list of sixteen recommendations for future studies. We believe that our recommendations, along with the use of a generic reporting standard, such as TRIPOD, STROBE, RECORD, or STARD, will increase the reproducibility and reusability of future studies and algorithms.

## Supplementary Information

**Additional file 1.**

**Additional file 2.**

**Additional file 3.**

## Data Availability

All data generated or analysed during the study are included in this published article and its supplementary information files.

## Abbreviations

ACL: Association for Computational Linguistics
ACM: Association for Computing Machinery
EHR: Electronic Health Record
FN: False Negatives
FP: False Positives
HPO: Human Phenotype Ontology
NLP: Natural Language Processing
PRISMA: Preferred Reporting Items for Systematic reviews and Meta-Analyses
TN: True Negatives
TP: True Positives

## References

[1] Ford E, Nicholson A, Koeling R, Tate AR, Carroll J, Axelrod L, et al. Optimising the use of electronic health records to estimate the incidence of rheumatoid arthritis in primary care: what information is hidden in free text? BMC Med Res Methodol. 2013;13. 10.1186/1471-2288-13-105 PMC3765394 23964710

[2] Rosenbloom ST, Denny JC, Xu H, Lorenzi N, Stead WW, Johnson KB (2011). Data from clinical notes: a perspective on the tension between structure and flexible documentation. J Am Med Informatics Assoc.

[3] Coorevits P, Sundgren M, Klein GO, Bahr A, Claerhout B, Daniel C (2013). Electronic health records: new opportunities for clinical research. J Intern Med.

[4] Danciu I, Cowan JD, Basford M, Wang X, Saip A, Osgood S (2014). Secondary use of clinical data: the Vanderbilt approach. J Biomed Inform.

[5] Price SJ, Stapley SA, Shephard E, Barraclough K, Hamilton WT. Is omission of free text records a possible source of data loss and bias in clinical practice research Datalink studies? A case-control study. BMJ Open. 2016;6. 10.1136/bmjopen-2016-011664 PMC4874123 27178981

[6] Gruber TR (1993). A translation approach to portable ontology specifications. Knowl Acquis.

[7] SNOMED International. SNOMED CT http://www.snomed.org/snomed-ct/five-step-briefing. Accessed 29 Jun 2020.

[8] Köhler S, Carmody L, Vasilevsky N, Jacobsen JOB, Danis D, Gourdine JP (2019). Expansion of the human phenotype ontology (HPO) knowledge base and resources. Nucleic Acids Res.

[9] Krasowski M, Schriever A, Mathur G, Blau J, Stauffer S, Ford B (2015). Use of a data warehouse at an academic medical center for clinical pathology quality improvement, education, and research. J Pathol Inform.

[10] Wu H, Toti G, Morley KI, Ibrahim ZM, Folarin A, Jackson R (2018). SemEHR: a general-purpose semantic search system to surface semantic data from clinical notes for tailored care, trial recruitment, and clinical research. J Am Med Inf Assoc.

[11] Shivade C, Malewadkar P, Fosler-Lussier E, Lai AM (2015). Comparison of UMLS terminologies to identify risk of heart disease using clinical notes. J Biomed Inform.

[12] Lingren T, Thaker V, Brady C, Namjou B, Kennebeck S, Bickel J (2016). Developing an algorithm to detect early childhood obesity in two tertiary pediatric medical centers. Appl Clin Inform.

[13] Ni Y, Kennebeck S, Dexheimer JW, McAneney CM, Tang H, Lingren T (2015). Automated clinical trial eligibility prescreening: increasing the efficiency of patient identification for clinical trials in the emergency department. J Am Med Informatics Assoc..

[14] Sun H, Depraetere K, De Roo J, Mels G, De Vloed B, Twagirumukiza M (2015). Semantic processing of EHR data for clinical research. J Biomed Inform.

[15] Kreimeyer K, Foster M, Pandey A, Arya N, Halford G, Jones SF (2017). Natural language processing systems for capturing and standardizing unstructured clinical information: a systematic review. J Biomed Inf.

[16] Gonzalez-Hernandez G, Sarker A, O’Connor K, Savova G (2017). Capturing the Patient’s perspective: a review of advances in natural language processing of health-related text. Yearb Med Inf.

[17] Jovanovic J, Bagheri E, Jovanović J, Bagheri E, Jovanovic J, Bagheri E (2017). Semantic annotation in biomedicine: the current landscape. J Biomed Semant.

[18] UK EQUATOR Centre. The EQUATOR Network. https://www.equator-network.org/. Accessed 29 Jun 2020.

[19] Ford E, Carroll JA, Smith HE, Scott D, Cassell JA (2016). Extracting information from the text of electronic medical records to improve case detection: a systematic review. J Am Med Informatics Assoc..

[20] Vuokko R, Makela-Bengs P, Hypponen H, Lindqvist M, Doupi P, Mäkelä-Bengs P (2017). Impacts of structuring the electronic health record: results of a systematic literature review from the perspective of secondary use of patient data. Int J Med Inform.

[21] Collins GS, Reitsma JB, Altman DG, Moons KGM, TRIPOD Group (2015). Transparent reporting of a multivariable prediction model for individual prognosis or diagnosis (TRIPOD): the TRIPOD statement. TRIPOD Group Circ.

[22] von Elm E, Altman DG, Egger M, Pocock SJ, Gøtzsche PC, Vandenbroucke JP (2008). The strengthening the reporting of observational studies in epidemiology (STROBE) statement: guidelines for reporting observational studies. J Clin Epidemiol.

[23] Benchimol EI, Smeeth L, Guttmann A, Harron K, Moher D, Peteresen I et al. The REporting of studies Conducted using Observational Routinely-collected health Data (RECORD) Statement. PLoS Med. 2015;12:1–22. 10.1371/journal.pmed.1001885 PMC4595218 26440803

[24] Bossuyt PM, Reitsma JB, Bruns DE, Gatsonis CA, Glasziou PP, Irwig L, et al. STARD 2015: an updated list of essential items for reporting diagnostic accuracy studies. BMJ. 2015;351:h5527. 10.1136/bmj.h5527 PMC4623764 26511519

[25] Moher D, Liberati A, Tetzlaff J, Altman DG, Altman D, Antes G et al. Preferred reporting items for systematic reviews and meta-analyses: The PRISMA statement. PLoS Med. 2009;6:1–6. PMC3090117 21603045

[26] The EndNote Team. EndNote. Philadelphia: Clarivate; 2013.

[27] Ouzzani M, Hammady H, Fedorowicz Z, Elmagarmid A. Rayyan—a web and mobile app for systematic reviews. Syst Rev. 2016;5:210. 10.1186/s13643-016-0384-4 PMC5139140 27919275

[28] Veritas Health Innovation. Covidence systematic review software. Melbourne: Veritas Health Innovation; 2020.

[29] Afshar M, Dligach D, Sharma B, Cai X, Boyda J, Birch S (2019). Development and application of a high throughput natural language processing architecture to convert all clinical documents in a clinical data warehouse into standardized medical vocabularies. J Am Med Inform Assoc.

[30] Alnazzawi N, Thompson P, Ananiadou S (2016). Mapping Phenotypic Information in Heterogeneous Textual Sources to a Domain-Specific Terminological Resource. PLoS One.

[31] Atutxa A, Perez A, Casillas A (2018). Machine Learning Approaches on Diagnostic Term Encoding with the ICD for Clinical Documentation. IEEE J Biomed Heal Informatics.

[32] Barrett N, Weber-Jahnke JH, Thai V (2013). Engineering natural language processing solutions for structured information from clinical text: extracting sentinel events from palliative care consult letters. Stud Health Technol Inform.

[33] Becker M, Bockmann B (2016). Extraction of UMLS(R) Concepts Using Apache cTAKES for German Language. Stud Health Technol Inform.

[34] Becker M, Kasper S, Böckmann B, Jöckel K-H, Virchow I (2019). Natural language processing of German clinical colorectal cancer notes for guideline-based treatment evaluation. Int J Med Inform.

[35] Bejan CA, Wei WQ, Denny JC (2015). Assessing the role of a medication-indication resource in the treatment relation extraction from clinical text. J Am Med Informatics Assoc.

[36] Castro E, Iglesias A, Martínez P, Castaño L (2010). Automatic Identification of Biomedical Concepts in Spanish-language Unstructured Clinical Texts. German Research Cent for Artificial, Intelligence - DFKI GmbH, Kaiserslautern, Germany Seattle, WA, USA: ACM.

[37] Catling F, Spithourakis GP, Riedel S (2018). Towards automated clinical coding. Int J Med Inform.

[38] Chapman WW, Fiszman M, Dowling JN, Chapman BE, Rindflesch TC (2004). Identifying respiratory findings in emergency department reports for biosurveillance using MetaMap. Medinfo.

[39] Chen J, Zheng J, Yu H (2016). Finding Important Terms for Patients in Their Electronic Health Records: A Learning-to-Rank Approach Using Expert Annotations. JMIR Med informatics.

[40] Chiaramello E, Pinciroli F, Bonalumi A, Caroli A, Tognola G (2016). Use of “off-the-shelf” information extraction algorithms in clinical informatics: A feasibility study of MetaMap annotation of Italian medical notes. J Biomed Inform.

[41] Chodey KP, Hu G (2016). Clinical text analysis using machine learning methods. 2016 IEEE/ACIS 15th International Conference on Computer and Information Science (ICIS).

[42] Chung J, Murphy S. Concept-value pair extraction from semi-structured clinical narrative: a case study using echocardiogram reports. AMIA Annu Symp Proc. 2005:131–5. PMC1560613 16779016

[43] Combi C, Zorzi M, Pozzani G, Moretti U, Arzenton E (2018). From narrative descriptions to MedDRA: automagically encoding adverse drug reactions. J Biomed Inform.

[44] de Bruijn B, Cherry C, Kiritchenko S, Martin J, Zhu X (2011). Machine-learned solutions for three stages of clinical information extraction: The state of the art at i2b2 2010. J Am Med Informatics Assoc.

[45] Deisseroth CA, Birgmeier J, Bodle EE, Kohler JN, Matalon DR, Nazarenko Y (2019). ClinPhen extracts and prioritizes patient phenotypes directly from medical records to expedite genetic disease diagnosis. Genet Med.

[46] Demner-Fushman D, Rogers WJ, Aronson AR (2017). MetaMap Lite: An evaluation of a new Java implementation of MetaMap. J Am Med Informatics Assoc.

[47] Divita G, Zeng QT, Gundlapalli AV, Duvall S, Nebeker J, Samore MH (2014). Sophia: A Expedient UMLS Concept Extraction Annotator. AMIA Annu Symp Proc.

[48] Duarte F, Martins B, Pinto CS, Silva MJ (2018). Deep neural models for ICD-10 coding of death certificates and autopsy reports in free-text. J Biomed Inform.

[49] Falis M, Pajak M, Lisowska A, Schrempf P, Deckers L, Mikhael S (2019). Ontological attention ensembles for capturing semantic concepts in ICD code prediction from clinical text.

[50] Ferrão JC, Janela F, Oliveira MD, HMG M (2013). Using Structured EHR Data and SVM to Support ICD-9-CM Coding. 2013 IEEE International Conference on Healthcare Informatics.

[51] Gerbier S, Yarovaya O, Gicquel Q, Millet A-L, Smaldore V, Pagliaroli V, et al. Evaluation of natural language processing from emergency department computerized medical records for intra-hospital syndromic surveillance. BMC Med Inform Decis Mak. 2011;11:50. 10.1186/1472-6947-11-50 PMC3158541 21798029

[52] Goicoechea Salazar JA, Nieto García MA, Laguna Téllez A, Canto Casasola VD, Rodríguez Herrera J, Murillo CF (2013). Development of an automated coding system to retrieve and analyze diagnostic information stored in hospital emergency department records. Emergencias.

[53] Hamid H, Fodeh SJ, Lizama AG, Czlapinski R, Pugh MJ, LaFrance WC (2013). Validating a natural language processing tool to exclude psychogenic nonepileptic seizures in electronic medical record-based epilepsy research. Epilepsy Behav.

[54] Hassanzadeh H, Kholghi M, Nguyen A, Chu K (2018). Clinical document classification using labeled and unlabeled data across hospitals. AMIA . Annu Symp proceedings AMIA Symp.

[55] Helwe C, Elbassuoni S, Geha M, Hitti E, Makhlouf OC (2017). CCS Coding of Discharge Diagnoses via Deep Neural Networks. German Research Cent for Artificial, Intelligence - DFKI GmbH, Kaiserslautern, Germany Seattle, WA, USA: ACM.

[56] Hersh W, Mailhot M, Arnott-Smith C, Lowe H (2001). Selective automated indexing of findings and diagnoses in radiology reports. J Biomed Inform.

[57] Hoogendoorn M, Szolovits P, Moons LMG, Numans ME (2015). Utilizing uncoded consultation notes from electronic medical records for predictive modeling of colorectal cancer. Artif Intell Med..

[58] Jindal P, Roth D (2013). Extraction of events and temporal expressions from clinical narratives. J Biomed Inform.

[59] Kang BY, Kim DW, Kim HG (2009). Two-phase chief complaint mapping to the UMLS metathesaurus in Korean Electronic Medical Records. IEEE Trans Inf Technol Biomed.

[60] Kersloot MGMG, Lau F, Abu-Hanna A, Arts DLDL, Cornet R (2019). Automated SNOMED CT concept and attribute relationship detection through a web-based implementation of cTAKES. J Biomed Semantics..

[61] König M, Sander A, Demuth I, Diekmann D, Steinhagen-Thiessen E (2019). Knowledge-based best of breed approach for automated detection of clinical events based on German free text digital hospital discharge letters. PLoS One.

[62] Li Q, Spooner SA, Kaiser M, Lingren N, Robbins J, Lingren T, et al. An end-to-end hybrid algorithm for automated medication discrepancy detection. BMC Med Inform Decis Mak. 2015;15:37. 10.1186/s12911-015-0160-8 PMC4427951 25943550

[63] Li F, Jin Y, Liu W, Rawat BPS, Cai P, Yu H (2019). Fine-tuning bidirectional encoder representations from transformers (BERT)-based models on large-scale electronic health record notes: an empirical study. JMIR Med informatics.

[64] Liu C, Ta CN, Rogers JR, Li Z, Lee J, Butler AM (2019). Ensembles of natural language processing systems for portable phenotyping solutions. J Biomed Inform.

[65] Lowe HJ, Huang Y, Regula DP (2009). Using a statistical natural language Parser augmented with the UMLS specialist lexicon to assign SNOMED CT codes to anatomic sites and pathologic diagnoses in full text pathology reports. AMIA Annu Symp Proc.

[66] Luo Y, Sohani AR, Hochberg EP, Szolovits P (2014). Automatic lymphoma classification with sentence subgraph mining from pathology reports. J Am Med Informatics Assoc.

[67] Meystre S, Haug PJ (2006). Natural language processing to extract medical problems from electronic clinical documents: performance evaluation. J Biomed Inform.

[68] Meystre SM, Thibault J, Shen S, Hurdle JF, South BR (2010). Automatically detecting medications and the reason for their prescription in clinical narrative text documents. Stud Health Technol Inform..

[69] Minard AL, Ligozat AL, Abacha AB, Bernhard D, Cartoni B, Deléger L (2011). Hybrid methods for improving information access in clinical documents: Concept, assertion, and relation identification. J Am Med Informatics Assoc.

[70] Mishra R, Burke A, Gitman B, Verma P, Engelstad M, Haendel MA (2019). Data-driven method to enhance craniofacial and oral phenotype vocabularies. J Am Dent Assoc.

[71] Nguyen AN, Truran D, Kemp M, Koopman B, Conlan D, O’Dwyer J (2018). Computer-assisted diagnostic coding: effectiveness of an NLP-based approach using SNOMED CT to ICD-10 mappings. AMIA . Annu Symp proceedings AMIA Symp.

[72] Oellrich A, Collier N, Smedley D, Groza T (2015). Generation of silver standard concept annotations from biomedical texts with special relevance to phenotypes. PLoS One.

[73] Patrick JD, Nguyen DHM, Wang Y, Li M (2011). A knowledge discovery and reuse pipeline for information extraction in clinical notes. J Am Med Informatics Assoc.

[74] Pérez A, Atutxa A, Casillas A, Gojenola K, Sellart Á (2018). Inferred joint multigram models for medical term normalization according to ICD. Int J Med Inform.

[75] Reátegui R, Ratté S (2018). Comparison of MetaMap and cTAKES for entity extraction in clinical notes. BMC Med Inform Decis Mak.

[76] Roberts K, Harabagiu SM (2011). A flexible framework for deriving assertions from electronic medical records. J Am Med Informatics Assoc.

[77] Rousseau JF, Ip IK, Raja AS, Valtchinov VI, Cochon L, Schuur JD (2019). Can automated retrieval of data from emergency department physician notes enhance the imaging order entry process?. Appl Clin Inform.

[78] Savova GK, Masanz JJ, Ogren PV, Zheng J, Sohn S, Kipper-Schuler KC (2010). Mayo clinical text analysis and knowledge extraction system (cTAKES): architecture, component evaluation and applications. J Am Med Informatics Assoc..

[79] Shoenbill K, Song Y, Gress L, Johnson H, Smith M, Mendonca EA. Natural language processing of lifestyle modification documentation. Health Informatics J. 2019:1460458218824742. 10.1177/1460458218824742 PMC6722039 30791802

[80] Sohn S, Clark C, Halgrim SR, Murphy SP, Chute CG, Liu H (2014). MedXN: An open source medication extraction and normalization tool for clinical text. J Am Med Informatics Assoc.

[81] Solti I, Aaronson B, Fletcher G, Solti M, Gennari JH, Cooper M, et al. Building an automated problem list based on natural language processing: lessons learned in the early phase of development. AMIA Annu Symp Proc. 2008;2008:687–91. PMC2655946 18999050

[82] Soriano IM, Peña JLC, Breis JTF, Román IS, Barriuso AA, Baraza DG (2019). Snomed2Vec: Representation of SNOMED CT Terms with Word2Vec. 2019 IEEE 32nd International Symposium on Computer-Based Medical Systems (CBMS).

[83] Soysal E, Wang J, Jiang M, Wu Y, Pakhomov S, Liu H (2018). CLAMP - a toolkit for efficiently building customized clinical natural language processing pipelines. J Am Med Informatics Assoc.

[84] Spasić I, Zhao B, Jones CB, Button K (2015). KneeTex: An ontology-driven system for information extraction from MRI reports. J Biomed Semantics.

[85] Strauss JA, Chao CR, Kwan ML, Ahmed SA, Schottinger JE, Quinn VP (2013). Identifying primary and recurrent cancers using a SAS-based natural language processing algorithm. J Am Med Informatics Assoc.

[86] Sung SF, Chen K, Wu DP, Hung LC, Su YH, Hu YH (2018). Applying natural language processing techniques to develop a task-specific EMR interface for timely stroke thrombolysis: A feasibility study. Int J Med Inform.

[87] Tchechmedjiev A, Abdaoui A, Emonet V, Zevio S, Jonquet C (2018). SIFR annotator: ontology-based semantic annotation of French biomedical text and clinical notes. BMC Bioinformatics.

[88] Ternois I, Escudie J-B, Benamouzig R, Duclos C (2018). Development of an automatic coding system for digestive endoscopies. Stud Health Technol Inform.

[89] Travers DA, Haas SW (2004). Evaluation of Emergency Medical Text Processor, a system for cleaning chief complaint text data. Acad Emerg Med.

[90] Tulkens S, Šuster S, Daelemans W (2019). Unsupervised concept extraction from clinical text through semantic composition. J Biomed Inform.

[91] Usui M, Aramaki E, Iwao T, Wakamiya S, Sakamoto T, Mochizuki M (2018). Extraction and standardization of patient complaints from electronic medication histories for Pharmacovigilance: natural language processing analysis in Japanese. JMIR Med informatics..

[92] Valtchinov VI, Lacson R, Wang A, Khorasani R (2019). Comparing Artificial Intelligence Approaches to Retrieve Clinical Reports Documenting Implantable Devices Posing MRI Safety Risks. J Am Coll Radiol.

[93] Wadia R, Akgun K, Brandt C, Fenton BT, Levin W, Marple AH (2018). Comparison of natural language processing and manual coding for the identification of cross-sectional imaging reports suspicious for lung Cancer. JCO Clin cancer informatics.

[94] Walker G, Soysal E, Xu H (2019). Development of a natural language processing tool to extract radiation treatment sites. Cureus..

[95] Xie X, Xiong Y, Yu PS, Zhu Y (2019). EHR Coding with Multi-scale Feature Attention and Structured Knowledge Graph Propagation. ACM.

[96] Xu H, Fu Z, Shah A, Chen Y, Peterson NB, Chen Q (2011). Extracting and integrating data from entire electronic health records for detecting colorectal cancer cases. AMIA Annu Symp Proc.

[97] Yadav K, Sarioglu E, Smith M, Choi HA (2013). Automated outcome classification of emergency department computed tomography imaging reports. Acad Emerg Med.

[98] Yao L, Mao C, Luo Y (2019). Clinical text classification with rule-based features and knowledge-guided convolutional neural networks. BMC Med Inform Decis Mak.

[99] Zeng Z, Espino S, Roy A, Li X, Khan SA, Clare SE (2018). Using natural language processing and machine learning to identify breast cancer local recurrence. BMC Bioinformatics.

[100] Zhang S, Elhadad N (2013). Unsupervised biomedical named entity recognition: Experiments with clinical and biological texts. J Biomed Inform.

[101] Zhou X, Han H, Chankai I, Prestrud A, Brooks A (2006). Approaches to Text Mining for Clinical Medical Records. German Research Cent for Artificial, Intelligence - DFKI GmbH, Kaiserslautern, Germany Seattle, WA, USA: ACM.

[102] Zhou L, Plasek JM, Mahoney LM, Karipineni N, Chang F, Yan X (2011). Using Medical Text Extraction, Reasoning and Mapping System (MTERMS) to process medication information in outpatient clinical notes. AMIA Annu Symp Proc.

[103] Zhou L, Lu Y, Vitale CJ, Mar PL, Chang F, Dhopeshwarkar N (2014). Representation of information about family relatives as structured data in electronic health records. Appl Clin Inform.

[104] Hassanzadeh H, Nguyen A, Koopman B (2016). Evaluation of Medical Concept Annotation Systems on Clinical Records.

[105] Matentzoglu N, Malone J, Mungall C, Stevens R (2018). MIRO: guidelines for minimum information for the reporting of an ontology. J Biomed Semantics.

[106] Beleites C, Neugebauer U, Bocklitz T, Krafft C, Popp J (2013). Sample size planning for classification models. Anal Chim Acta.

[107] Yang Q, Liu Y, Chen T, Tong Y (2019). Federated machine learning: concept and applications. ACM Trans Intell Syst Technol.

[108] Sokolova M, Lapalme G (2009). A systematic analysis of performance measures for classification tasks. Inf Process Manag.

