# Supplementary material for: Natural language processing algorithms for mapping clinical text fragments onto ontology concepts: a systematic review and recommendations for future studies
Source: J Biomed Semantics. 2020 Nov 16;11:14. doi: 10.1186/s13326-020-00231-z (PMC7670625; doi:10.1186/s13326-020-00231-z)
Supplement: Supplementary file 2 — Additional file 2. [file 13326_2020_231_MOESM2_ESM.docx]

| **Database** | **#** | **Query** |
| --- | --- | --- |
| **MEDLINE** | 1 | (("Natural Language Processing"[tiab] OR "Natural Language Processing"[MeSH] OR "Medical Language Processing"[tiab]) AND (Electronic Health Record*[tiab] OR EHR[tiab] OR EMR[tiab] OR electronic medical record*[tiab] OR "Electronic Health Records"[MeSH] OR report[tiab] OR chart[tiab] OR reports[tiab] OR charts[tiab] OR "clinical notes"[tiab] OR "clinical text"[tiab] OR "medical notes"[tiab] OR "medical text"[tiab])) AND (ontolog*[tiab] OR concept*[tiab] OR encod*[tiab] OR annotat*[tiab] OR "code"[tiab] OR "coding"[tiab] OR "Vocabulary, Controlled"[MeSH]) |
| **Scopus** | 1 | ( ( TITLE-ABS-KEY-AUTH ( "Natural Language Processing" ) OR TITLE-ABS-KEY-AUTH ( "Medical Language Processing" )  ) AND ( TITLE-ABS-KEY-AUTH ( Electronic Health Record* ) OR TITLE-ABS-KEY-AUTH ( ehr ) OR TITLE-ABS-KEY-AUTH ( emr ) OR TITLE-ABS-KEY-AUTH ( electronic medical record* ) OR TITLE-ABS-KEY-AUTH ( report ) OR TITLE-ABS-KEY-AUTH ( chart ) OR TITLE-ABS-KEY-AUTH ( reports ) OR TITLE-ABS-KEY-AUTH ( charts ) OR TITLE-ABS-KEY-AUTH ( "clinical notes" ) OR TITLE-ABS-KEY-AUTH ( "clinical text" ) OR TITLE-ABS-KEY-AUTH ( "medical notes" ) OR TITLE-ABS-KEY-AUTH ( "medical text" ) ) AND ( TITLE-ABS-KEY-AUTH ( ontolog* ) OR TITLE-ABS-KEY-AUTH ( concept* ) OR TITLE-ABS-KEY-AUTH ( encod* ) OR TITLE-ABS-KEY-AUTH ( annotat* ) OR TITLE-ABS-KEY-AUTH ( "code" ) OR TITLE-ABS-KEY-AUTH ( "coding" ))) AND ( LIMIT-TO ( DOCTYPE , "ar" ) ) AND ( LIMIT-TO ( SUBJAREA , "MEDI" ) OR LIMIT-TO ( SUBJAREA , "HEAL" ) OR LIMIT-TO ( SUBJAREA , "NURS" ) ) AND ( LIMIT-TO ( LANGUAGE , "English" ) ) |
| **IEEE** | 1 | (("Natural Language Processing" OR "Medical Language Processing") AND (Electronic Health Record OR Electronic Health Records OR EHR OR EMR OR electronic medical record* OR report OR chart OR reports OR charts OR "clinical notes" OR "clinical text" OR "medical notes" OR "medical text") AND (ontolog* OR concept* OR encod* OR annotat* OR "code" OR "coding") ) |
| **Embase** | 1 | (("Natural Language Processing" OR "Medical Language Processing") AND (Electronic Health Record* OR EHR OR EMR OR electronic medical record* OR report OR chart OR reports OR charts OR "clinical notes" OR "clinical text" OR "medical notes" OR "medical text") AND (ontolog* OR concept* OR encod* OR annotat* OR "code" OR "coding" ) ).mp. [mp=title, abstract, heading word, drug trade name, original title, device manufacturer, drug manufacturer, device trade name, keyword, floating subheading word] |
| **ACL** | 1 | ("Electronic Medical Record" OR "Electronic Medical Records") AND ("Natural Language Processing" OR "Medical Language Processing") AND (ontolog* OR concept* OR encod* OR annotat* OR "code" OR "coding") site:aclweb.org |
|  | 2 | ("Electronic Health Record" OR "Electronic Health Records") AND ("Natural Language Processing" OR "Medical Language Processing") AND (ontolog* OR concept* OR encod* OR annotat* OR "code" OR "coding") site:aclweb.org |
|  | 3 | ("Clinical note" OR "Clinical notes") AND ("Natural Language Processing" OR "Medical Language Processing") AND (ontolog* OR concept* OR encod* OR annotat* OR "code" OR "coding") site:aclweb.org |
|  | 4 | ("Clinical text" OR "Clinical texts") AND ("Natural Language Processing" OR "Medical Language Processing") AND (ontolog* OR concept* OR encod* OR annotat* OR "code" OR "coding") site:aclweb.org |
|  | 5 | ("Medical note" OR "Medical notes") AND ("Natural Language Processing" OR "Medical Language Processing") AND (ontolog* OR concept* OR encod* OR annotat* OR "code" OR "coding") site:aclweb.org |
|  | 6 | ("Medical text" OR "Medical texts") AND ("Natural Language Processing" OR "Medical Language Processing") AND (ontolog* OR concept* OR encod* OR annotat* OR "code" OR "coding") site:aclweb.org |
| **ACM** | 1 | ("Electronic Medical Record" OR "Electronic Medical Records") AND ("Natural Language Processing" OR "Medical Language Processing") AND (ontolog* OR concept* OR encod* OR annotat* OR "code" OR "coding") site:dl.acm.org |
|  | 2 | ("Electronic Health Record" OR "Electronic Health Records") AND ("Natural Language Processing" OR "Medical Language Processing") AND (ontolog* OR concept* OR encod* OR annotat* OR "code" OR "coding") site:dl.acm.org |
|  | 3 | ("Clinical note" OR "Clinical notes") AND ("Natural Language Processing" OR "Medical Language Processing") AND (ontolog* OR concept* OR encod* OR annotat* OR "code" OR "coding") site:dl.acm.org |
|  | 4 | ("Clinical text" OR "Clinical texts") AND ("Natural Language Processing" OR "Medical Language Processing") AND (ontolog* OR concept* OR encod* OR annotat* OR "code" OR "coding") site:dl.acm.org |
|  | 5 | ("Medical note" OR "Medical notes") AND ("Natural Language Processing" OR "Medical Language Processing") AND (ontolog* OR concept* OR encod* OR annotat* OR "code" OR "coding") site:dl.acm.org |
|  | 6 | ("Medical text" OR "Medical texts") AND ("Natural Language Processing" OR "Medical Language Processing") AND (ontolog* OR concept* OR encod* OR annotat* OR "code" OR "coding") site:dl.acm.org |
