# Supplementary material for: Natural language processing algorithms for mapping clinical text fragments onto ontology concepts: a systematic review and recommendations for future studies
Source: J Biomed Semantics. 2020 Nov 16;11:14. doi: 10.1186/s13326-020-00231-z (PMC7670625; doi:10.1186/s13326-020-00231-z)
Supplement: Supplementary file 3 — Additional file 3. [file 13326_2020_231_MOESM3_ESM.docx]

| **Characteristics** |  | **TRIPOD** | **STROBE** | **RECORD** | **STARD** |
| --- | --- | --- | --- | --- | --- |
| **Title and abstract** | | | | | |
| Title |  | 1 | 1 a | 1.1-1.3 | 1 |
| Abstract |  | 2 | 1 b | 1.1-1.3 | 2 |
| **Publication** | | | | | |
| Year of publication |  | - | - | - | - |
| Country |  | - | - | - | - |
| **Introduction** | | | | | |
| Objectives |  | 3 b | 3 | - | 4 |
| **Methods** | | | | | |
| Study design | Evaluation methods  Used tools  Used Terminology systems | 4 a | 4 | - | 5 |
| Setting |  | 5 a | 5 | - | - |
| Sample size | Size of dataset | 8 | 10 | - | 18 |
| *Statistical analysis methods* | | | | | |
| Measures to assess performance |  | 10 d | 12 e | - | 14 |
| Reference standard |  | - | - | - | 10 b, 11 |
| **Results** | | | | | |
| Characteristics | Language of the text | 13 b | - | 13.1 | 20, 21 a, b |
| **Discussion** | | | | | |
| Validation |  | 19 a | - | - | - |
| Generalisability | External validity | - | 21 | - | 26 |
| Operational use |  | - | - | - | - |
| **Other** | | | | | |
| Source code |  | - | - | 22.1 | - |
